# Association between protoporphyrin IX and sarcopenia: a cross sectional study

**DOI:** 10.1186/s12877-021-02331-6

**Published:** 2021-06-26

**Authors:** Chia-Chun Kao, Zhe-Yu Yang, Wei-Liang Chen

**Affiliations:** 1 Division of Family Medicine, Department of Family and Community Medicine, Tri-Service General Hospital; and School of Medicine, National Defense Medical Center, Taiwan, Republic of China; 2 Division of Geriatric Medicine, Department of Family and Community Medicine, Tri-Service General Hospital; and School of Medicine, National Defense Medical Center, Taiwan, Republic of China; 3 Department of Biochemistry, National Defense Medical Center, Taiwan, Republic of China

**Keywords:** Protoporphyrin IX, Sarcopenia, Mitochondria, Heme oxygenase-1

## Abstract

**Background:**

According to the European Working Group on Sarcopenia in Older People (EWGSOP), the diagnosis of sarcopenia primarily focused on low muscle strength with the detection of low muscle quality and quantity as confirming index. Many studies had identified mitochondrial dysfunction as one of the multifactorial etiologies of sarcopenia. Yet, no study had investigated the role of biosynthetic pathway intermediate, which was found in mitochondria, in the development of sarcopenia. This study aimed to examine the association between protoporphyrin IX (PPIX) and components of sarcopenia.

**Method:**

The present study enrolled 1172 participants without anemia between 1999 to 2002 from the National Health and Nutrition Examination Survey (NHANES) database. We employed the multivariable-logistic regression model to examine the relationship between PPIX and sarcopenia. Covariate adjustments were designated to each of the three models for further analysis of the relationship.

**Results:**

In the unadjusted model, PPIX was significantly associated with sarcopenia (OR = 3.910, 95% CI = 2.375, 6.439, *P* value < 0.001). The significance persisted after covariate adjustments as observed in the fully adjusted model (OR = 2.537, 95% CI = 1.419, 4.537, *P* value = 0.002).

**Conclusions:**

The findings of this study suggested statistically significant association between PPIX and sarcopenia. Our study disclosed the potential of PPIX as a valuable indicator of sarcopenia.

**Supplementary Information:**

The online version contains supplementary material available at 10.1186/s12877-021-02331-6.

## Introduction

Sarcopenia is a generalized skeletal muscle disorder that involves the progressive loss of muscle mass and function [1], leading to substantial functional decline, development of chronic diseases, disability, and frailty [2–5]. The European Working Group on Sarcopenia in Older People (EWGSOP) developed a set of clinical guidelines and consensus diagnostic criteria, which emphasized on the presence of low muscle strength and low muscle mass as the definition of sarcopenia with physical performance being only a severity gradient [6]. Sarcopenia that is largely related to age in the absence of other identifiable cause is defined as primary sarcopenia. Sarcopenia is considered ‘secondary’ when factors other than age are identified [6]. The mechanisms associated with the development and progression of sarcopenia include endocrine dysfunction [7], neuro-degenerative diseases [8], malnutrition [9], cachexia [10], aging [10], disuse [11], and cellular dysfunctions [12]. Studies had pointed out the significances of mitochondrial dysfunction in the pathogenesis of sarcopenia [12–15]. However, the associated pathway of mitochondrial dysfunction leading up to sarcopenia is still unclear. More researches and clinical studies on mitochondrial dysfunction induced sarcopenia are required.

Several researchers have outlined that decreased hemoglobin is linked to sarcopenia [16–18]. While anemia is evidently associated with sarcopenia, investigation regarding non-anemic individuals with sarcopenia development is warranted. We suspect that the heme biosynthetic pathway, which takes place in mitochondria, responsible for the generation of hemoprotein constituent is related to the development of sarcopenia. Protoporphyrin IX (PPIX) is the final intermediate in the heme biosynthetic pathway. PPIX is a heterocyclic organic compound that exhibits biological functions by chelating to transition metals to form metalloporphyrins [19]. A mice study highlighted accumulating PPIX mediated alterations in mitochondrial membrane potentials and formation of fragmented mitochondria in hepatocytes [20]. PPIX is commonly converted to heme by chelating to iron under the catalyzation of an enzyme known as ferrochelatase [19]. Nevertheless, the heme biosynthetic pathway could get interrupted under low iron concentration and lead toxicity. This results in the substitution of other transitional metals, particularly zinc, for iron during the chelation, causing an increase in zinc PPIX level [21]. Taken together, PPIX plays a critical role as an intermediate of heme biosynthetic pathway in which a sustained homeostasis is required.

In this study, we hypothesize that alterations in serum PPIX concentration may play a role in the pathogenesis of sarcopenia. As no previous studies had investigated the association between PPIX and sarcopenia, we conducted a cross-sectional study on a group of nationally representative United States adult population to examine this issue.

## Materials and methods

### Ethics statement

The present study obtained data from the National Health and Nutrition Examination Survey (NHANES) [22, 23]. NHANES is a cross-sectional survey that collects information on demographic, clinical, behavioral, dietary, social, and laboratory data from non-institutionalized individuals in the United States. The survey was performed by the National Center for Health Statistics (NCHS) and approved by the NCHS Institutional Review Board (IRB). All informed consents had been obtained from the eligible subjects before initiating data collection and NHANES health examinations.

### Study sample

**Fig. 1:**
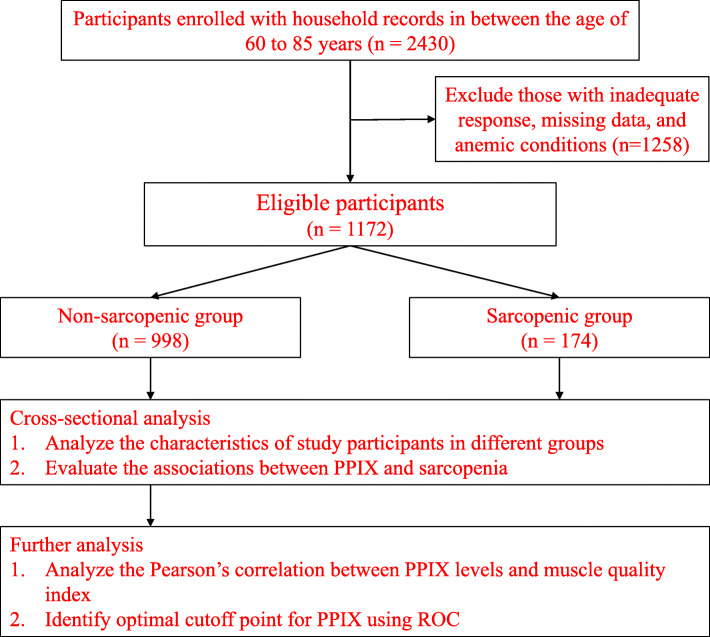
Flow chart representing the steps of analysis performed in the study

The study sample was collected from the NHANES database from 1999 to 2002. The interviews were conducted by trained examiners and consisted demographic information such as gender, age, race/ethnicity, education level, smoking history, and individual comorbidities. Physical examination was conducted at the Mobile Examination Center (MEC). According to the flowchart of the study in Fig. 1, we included a total of 2430 participants who had household records and excluded 1258 participants with missing data, inadequate interview responses, and anemic conditions. The cutoff points used to determine anemia were hemoglobin concentrations less than 13 g/dL for men and 12 g/dL for women [24]. The population surveyed in the current study were in between the age of 60 to 85 years. The demographic sample included 1172 eligible participants (*n* = 626 men; *n* = 546 women) as shown in Table 1. The survey was conducted in the United States between 1999 and 2002 by the NCHS of the Centers for Disease Control and Prevention using a stratified, multistage, and clustered probability sample design. Further details on the survey could be accessed on the NHANES website [25, 26].

**Table 1:** Characteristics of study participants

Characteristics of study participants	Non-sarcopenic group	Sarcopenic group	Total	***P*** value
	(n = 998)	(n = 174)	(***n*** = 1172)	
**Continuous variables** ^**a**^				
**Age at screening**	64.00 (14.00)	70.00 (18.00)	65.00 (14.00)	< 0.001
**Body mass index** **(kg/m**^**2**^**)**	28.68 (5.94)	22.52 (3.32)	27.81 (6.46)	< 0.001
**Protoporphyrin** **(μg/dL RBC)**	47.00 (18.00)	50.00 (22.00)	48.00 (19.00)	< 0.001
**Hemoglobin** **(g/dL)**	14.75 (1.70)	14.35 (1.60)	14.50 (1.70)	< 0.001
**Comorbidity**	0.00 (1.00)	0.00 (1.00)	0.00 (1.00)	< 0.001
**Average peak force (Newtons)**	281.30 (134.33)	207.50 (112.90)	270.10 (132.00)	< 0.001
**Skeletal muscle index (kg/m**^**2**^**)**	7.60 (1.81)	5.61 (1.52)	7.31 (1.98)	< 0.001
**Gait speed (m/s)**	1.00 (0.29)	0.96 (0.33)	0.99 (0.29)	< 0.001
**Categorical variables** ^**b**^ **(%)**				
**Race/ethnicity**	234 (23.4)	34 (19.5)	268 (22.9)	0.259
**Gender**	535 (53.6)	91 (52.2)	626 (53.4)	0.750
**Smoking**	548 (54.9)	108 (62.1)	656 (56.0)	0.072
**Education level past high school**	578 (57.9)	97 (56.1)	675 (57.6)	0.650
**Arthritis**	391 (39.2)	60 (34.5)	451 (38.5)	0.240
**Congestive heart failure**	39 (3.9)	12 (6.9)	51 (4.4)	0.075
**Coronary heart disease**	61 (6.1)	15 (8.6)	76 (6.5)	0.215
**Angina**	61 (6.1)	14 (8.0)	75 (6.4)	0.337
**Heart attack**	62 (6.2)	19 (10.9)	81 (6.9)	0.024
**Stroke**	6 (0.6)	2 (1.1)	8 (0.7)	0.418
**Emphysema**	23 (2.3)	13 (7.5)	36 (3.1)	< 0.001

*BMI* body mass index; *RBC* red blood cell

^a^ Values were expressed as median (interquartile range)

^b^ Values in the categorical variables were expressed as number (%)

The percentages of race/ethnicity, gender, and smoking represent Mexican American, men, and smokers respectively

### EWGSOP guideline

The definition and diagnostic criteria of sarcopenia in the present research was based on the revised EWGSOP guideline [6]. The diagnosis of sarcopenia was based on the documentation of low muscle strength and low muscle mass. Measurements of muscle strength included handgrip strength and knee flexion/extension techniques. The cutoff points for knee extension strength for men and women were respectively, 56.1 and 38.1 kg for one-repetition maximum as reported by Abdalla et al. [27]. This is equivalent to 550.15 and 373.63 Newtons for men and women. The use of knee extension strength in the evaluation of muscle strength was approved by the EWGSOP guideline. The EWGSOP guideline advised utilizing imaging modalities such as computed tomography (CT), magnetic resonance imaging (MRI), and dual energy X-ray absorptiometry (DXA) to estimate muscle mass or lean body mass. Low muscle mass was defined as skeletal muscle index (SMI) less or equal to 7.0 appendicular skeletal muscle mass (ASM) per height squared (kg/m^2^) for men and 5.5 kg/m^2^ for women [28]. Gait speed was a reliable and widely used test to evaluate physical performance. Low physical function or low gait speed was defined as walking speed ≤0.8 m/s.

### Measurement: muscle quality index

The standard protocol used to measure the muscle strength and gait speed of participants were available in the NHANES documentation [25, 26]. Participants with medical histories of major surgeries within 6 weeks, knee surgery, severe back pain, brain aneurysm, and stroke were excluded from the exam. The muscle strengths of participants were measured using A Kin Com MP dynamometer manufactured by Chattanooga Group, Inc., Chattanooga, TN. The peak torques of participants’ knee extensor strengths of the quadriceps were measured at one speed (60 degrees/second) and documented in Newtons. Participants’ habitual gait speed was measured on a 20 ft long test track set up in the MEC. All participants were required to walk at their habitual pace and timed using a hand-held stopwatch. All examinations were conducted by certified health technicians.

The muscle mass was estimated using the DXA. The DXA scans provide measurements of various body components including the bone and soft tissue. Female participants with positive urine pregnancy test or self-reported pregnancy were not permitted to undergo the DXA scan. Participants with weight over 300 pounds or height over six feet, five inches were excluded from the DXA examination. The DXA scan was conducted using the Hologic QDR-4500A fan-beam densitometer (Hologic, Inc., Bedford, Massachusetts) and Hologic software version 8.26:a3* by certified radiology technologists. The DXA scan was performed with the participants lying supine on the tabletop with feet in neutral position and hands flat by their side. Participants undergoing the examination were scanned with an x-ray of extremely low radiation exposure at less than 10 uSv. The scan acquired two low-dose x-ray images at different average energies to distinguish both bone from soft tissue and the percentage of fat in soft tissue when bone wasn’t present. The DXA scan measured the ASM, which involved lean soft tissue of arms and legs without bone mineral content. Specific details on the measurement could be attained from the NHANES documentation [25, 26].

### Measurement: PPIX

The measurement of PPIX involved extracting PPIX from EDTA-whole blood into a mixture of ethyl acetate-acetic acid and then back-extracted into diluted hydrochloric acid. In the aqueous form, the PPIX was measured at excitation wavelength of 404 nm and emission wavelength of 658 nm. The measurement of PPIX concentration was expressed in μg/dL of packed red blood cells (RBC) after correction for hematocrit in individual specimen. Specific details of the measurement could be obtained from the NHANES documentation [25, 26].

### Measurement: covariates

The demographic information concerning variables, such as race/ethnicity [29], sex [29–31], age, and medical history, including arthritis, congestive heart failure (CHF), coronary heart disease (CHD), angina, heart attack, stroke, and emphysema, were acquired from self-reported data. Participants’ cigarette use was recorded by asking the question, “Have you ever smoked cigarettes?” [32]. Participants’ education level was recorded by asking the question, “What is the highest grade or level of school you have completed or the highest degree have received?” [31]. Laboratory data such as body mass index (BMI) [31, 33], and hemoglobin [34] were analyzed in our study. Specific details on the measurement could be attained from the NHANES documentation [25, 26].

### Statistical analysis

All statistical analysis of this study was performed using SPSS (Version 18.0 for Windows, SPSS, Inc., Chicago, IL, USA). Continuous data were indicated by their median and interquartile range (IQR). Categorical data were recorded by their frequency counts and percentages. The chi-square test and analysis of variance (ANOVA) were applied to categorical variables and continuous variables, respectively. The distributions of PPIX levels were normalized using natural logarithm transformation. Odds ratios (OR) were calculated using logistic regression to evaluate the intensity of the relationship between PPIX and sarcopenia. To test the robustness of the primary result, we conducted a sensitivity analysis by dividing participants into quartiles of PPIX concentrations as shown in Additional file 1: Table S1. The present study further examined the intensity of the relationship between PPIX levels and strength/SMI/physical performance by investigating the Pearson correlation coefficients. We interpreted the association between PPIX and components of sarcopenia, using multivariable-linear regression models designed with progressive degrees of modification. Covariate adjustments were investigated by the following extended-model linear regressions: Model 1 was unadjusted; Model 2 was adjusted for race/ethnicity, sex, and age; Model 3 = Model 2 + BMI, comorbidity, smoking, education level, and hemoglobin. A receiving operating characteristic (ROC) curve analysis was used to examine the optimal cutoff values of PPIX.

## Results

### Study sample characteristics

The present study included a total of 1172 participants. The characteristics of eligible study participants were shown in Table 1. Based on the EWGSOP definition of sarcopenia, we divided participants into non-sarcopenic group (*n* = 998) and sarcopenic group (*n* = 174) [6]. According to our analysis, 190 participants had low muscle strength, 174 participants had low SMI, and 230 participants had low gait speed. All continuous variables observed showed statistical significance (*P* value < 0.001). The median age for the non-sarcopenic group and sarcopenic group were 64.00 (IQR = 14.00) and 70.00 (IQR = 18.00), respectively. It was also observed that the median PPIX concentration was higher for sarcopenic group at 50.00 (IQR = 22.00) compared to 47.00 (IQR = 18.00) in the non-sarcopenic group. Subjects with older age, higher BMI, increased PPIX concentrations, and more comorbidities tended to display signs of declined muscle quality including muscle strength, SMI, and gait speed.

### Association between PPIX and sarcopenia

In Table 2, we examined the intensity of the association between PPIX and sarcopenia by performing logistic regression. In Model 1, PPIX was significantly associated with sarcopenia (OR = 3.910, 95% CI = 2.375, 6.439, *P* value < 0.001). The significance persisted after covariate adjustments as observed in Model 2 (OR = 2.633, 95% CI = 1.520, 4.563, P value = 0.001) and Model 3 (OR = 2.537, 95% CI = 1.419, 4.537, P value = 0.002).

**Table 2:** Result of logistic regression predicting the presence of sarcopenia by protoporphyrin IX

		Models ^**a**^		
		Model ^**a**^ 1	Model ^**a**^ 2	Model ^**a**^ 3
**Sarcopenia**	**Odds ratio (95% CI)** ***P*** **value**	3.910 (2.375, 6.439) < 0.001	2.633 (1.520, 4.563) 0.001	2.537 (1.419, 4.537) 0.002

*BMI* body mass index; *CI* confidence interval; *OR* odds ratio

^a^ Adjusted covariates:

Model 1 = Unadjusted

Model 2 = Model 1 + age, sex, race/ethnicity

Model 3 = Model 2 + BMI, comorbidities, smoking, education level, hemoglobin

**Fig. 2:**
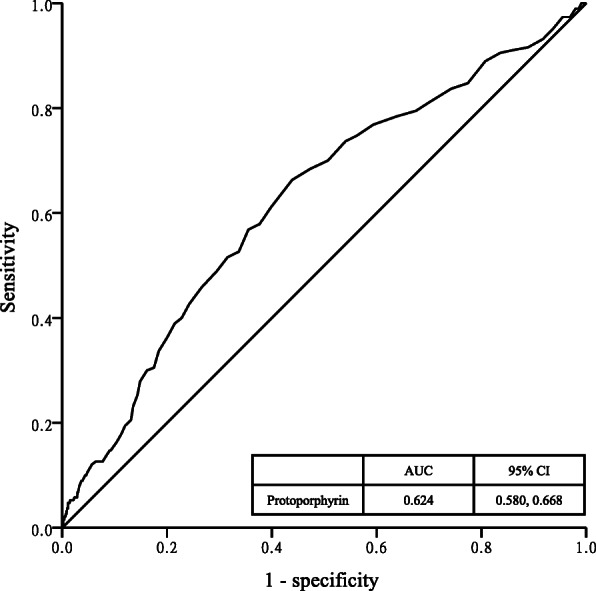
Receiver operating characteristic curve analysis

By using ROC curve analysis, Fig. 2 highlighted the optimal cutoff point for PPIX in the development of sarcopenia. The area under ROC (AUROC) value was 0.624 (95% CI = 0.580, 0.668). An optimal cutoff value of 48.50 μg/dL RBC were determined using maximal Youden’s index with sensitivity of 0.663 and specificity of 0.561.

### Sensitivity analysis

Sensitivity analysis using logistic regression to compare the analysis of sarcopenic and non-sarcopenic groups and quartiles of PPIX showed similar results for the association between PPIX and sarcopenia. Additional file 1: Table S2 was a logistic regression analysis displaying the association between quartiles of PPIX and components of sarcopenia. In the unadjusted model, we observed that the higher quartiles of PPIX concentration were significantly associated with low muscle strength and low gait speed in comparison to the lowest quartile of PPIX concentration. The fully adjusted model showed that the odds of participants with the highest quartile of PPIX concentrations would have low muscle strength and low SMI were 1.934 (1.174 ± 3.185) and 2.258 (1.172 ± 4.349) times greater than participants with the lowest quartile of PPIX concentrations. This result was similar to the result derived in Table 2 in which PPIX was significantly associated with sarcopenia before and after covariate adjustments.

### Correlation between PPIX and muscle quality index

In Table 3, the correlation coefficients between PPIX and muscle strength, SMI, and gait speed were − 0.182, − 0.123, and − 0.131 respectively. Table 4 showed the multivariate adjusted linear analysis of the association between PPIX and muscle quality index. The association between PPIX and muscle strength (β = − 69.413, 95% CI = − 89.981, − 49.846), SMI (β = − 0.634, 95% CI = − 0.898, − 0.370), and gait speed (β = 0.160 95% CI = 0.085, 0.235) in the unadjusted model revealed statistical significance (*P* value < 0.001). Similar trend in the fully adjusted model were observed where β coefficients for muscle strength, SMI, and gait speed were − 11.848 (95% CI = − 28.203, 4.507), − 0.186 (95% CI = − 0.309, − 0.064), and 0.048 (95% CI = − 0.025, 0.121). However, only the association between PPIX and skeletal muscle index revealed statistical significance (P value = 0.003).

**Table 3:** Pearson correlation coefficient between protoporphyrin IX and muscle quality index

Variables	Average peak force (Newtons)		Skeletal muscle index		Gait speed	
**Protoporphyrin** **(μg/dL RBC)**	**Correlation**	***P*** **value**	**Correlation**	***P*** **value**	**Correlation**	***P*** **value**
	−0.182	< 0.001	− 0.123	< 0.001	− 0.131	< 0.001

**Table 4:** Association between protoporphyrin IX and muscle quality index

	Muscle quality index					
	Average peak force (Newtons)		Skeletal muscle index		Gait speed	
**Models** ^**a**^	**β**^**b**^ **(95% CI)**	***P*** **Value**	**β**^**b**^ **(95% CI)**	***P*** **Value**	**β**^**b**^ **(95% CI)**	***P*** **Value**
**Model** ^**a**^ **1**	−69.413 (−88.981, −49.846)	< 0.001	− 0.634 (− 0.898, − 0.370)	< 0.001	0.160 (0.085, 0.235)	< 0.001
**Model** ^**a**^ **2**	−15.155 (−31.471, 1.161)	0.069	0.162 (− 0.052, 0.376)	0.137	0.080 (0.006, 0.154)	0.034
**Model** ^**a**^ **3**	−11.848 (−28.203, 4.507)	0.155	− 0.186 (− 0.309, − 0.064)	0.003	0.048 (− 0.025, 0.121)	0.200

*BMI* body mass index; *CI* confidence interval

^a^ Adjusted covariates

Model 1 = Unadjusted

Model 2 = Model 1 + age, sex, race/ethnicity

Model 3 = Model 2 + BMI, comorbidity, smoking, education level, hemoglobin

^b^ β coefficients were interpreted as change of protoporphyrin IX for each increase or decrease in muscle quality index

### Association between PPIX and components of sarcopenia

Under low serum iron concentrations, PPIX was positively associated with low muscle strength and low muscle mass as shown in Additional file 1: Table S3. The pattern of association with low muscle strength and low muscle mass persisted after covariate adjustments for race/ethnicity, gender, age, BMI, comorbidity, smoking, education level, and hemoglobin concentration. In study participants with normal serum iron concentrations, PPIX maintained significant positive correlations with low muscle strength. However, no strong relationship was drawn between PPIX and low muscle mass in participants with normal serum iron concentrations. Our results indicated that the associations in the normal serum iron group could not fulfill the EWGSOP criteria. In contrast, associations in the low iron group was consistent with the sarcopenia definition of the EWGSOP guideline.

## Discussion

The present study closely inspected the relationship between the PPIX concentration in a nationally representative sample of the United States adult population and the components of sarcopenia. Our results indicated strong relationships between PPIX concentrations and sarcopenia. The same correlations in participants with low serum iron concentrations were also observed. To the best of our knowledge, the present study was the first to investigate the role of PPIX in predicting sarcopenia in a group of 1172 males and females.

The development of sarcopenia is multicausal and remains controversial owing to its complex nature in pathogenesis. Current studies had discovered multiple factors that represent strong associations with the development of sarcopenia [35–37]. The reduction in mitochondrial biogenesis and its respective cellular changes were recognized as major contributing factors in the progression of sarcopenia [14, 38]. A decline in the functions of mitochondria was found to be associated with pathologic events such as Type 2 diabetes and Alzheimer’s disease, both of which were common in the geriatric population [39].

Mitochondrial biogenesis is responsible for the clearance of damaged mitochondria and prompts the generation of new mitochondria [40]. The process of mitochondrial biogenesis is prone to the destructive effects of toxins and environmental exposures [41]. Heme oxygenase-1 (HO-1), on the other hand, plays a protective role in the elimination of dysfunctional mitochondria and the stimulation of mitochondrial biogenesis [42, 43]. Recent studies by Takanche stressed the upregulating effects HO-1 had on antioxidant gene and mitochondrial biogenesis [40, 44]. An animal study on rat by Chen et al. explained the role of HO-1 in the expression of miR-27b, which increased mitochondrial biogenesis and suppressed systemic inflammation [45]. We speculate that HO-1 inhibitor zinc PPIX, which forms under the lack of iron reservoir, may implicate the cause behind the positive correlation between PPIX and components of sarcopenia. While increased expression of miR-27b mediated cytoprotective modulation in mitochondria, the coadministration of zinc PPIX reversed the regenerating activities of HO-1 [46]. Yu et al. also observed the similar inhibitory effects of zinc PPIX on the attenuation of mitochondrial biogenesis and anti-inflammation induced by HO-1 and Tetrahydroxystilbene glucoside [47]. Taken together, zinc PPIX entails sarcopenia via inducing inhibition on mitochondrial biogenesis. These discoveries were consistent with our finding, which illustrated positive association between PPIX concentration and components of sarcopenia. Our results also indicated the equivalent association under low serum iron concentration, supporting the formation of zinc PPIX and its subsequent inhibitory activities.

In addition to the augmentation in mitochondrial biogenesis, HO-1 had also been found to exhibit anti-inflammatory and neuroprotective properties in rats [48, 49]. Yu et al. discovered in their experiments that HO-1 inhibited muscle fiber atrophy through suppressing proinflammatory cytokines and downregulating specific enzyme activation [48]. Although they did not observe effects of zinc PPIX on HO-1 expression, they did recognize suppressed HO-1 activation caused by zinc PPIX. Khan et al., in their animal models, demonstrated the therapeutic effects of increased HO-1 in neurodegenerative disorders such as Alzheimer disease and Parkinson disease [49]. Their study also identified tin PPIX, also a HO-1 inhibitor, as the reversal agent of the cytoprotective effects of HO-1. According to accumulating studies, the buildup of PPIX caused degenerative mitochondrial function, inflammation, and reduced cytoprotection. The accumulation of PPIX and its inhibitory nature implicates intrinsic role in the development of sarcopenia.

The present study has several strengths. While the screening involved in sarcopenia has been clearly identified in the EWGSOP guideline, a predictive biomarker for sarcopenia is lacking. Through our research, we offered PPIX as a highly potential biomarker in the prognostic prediction of sarcopenia. Moreover, the study included a large sample of older adults with racially mixed sample in the demographics to examine the associations. A defined biomarker such as PPIX proposed in the current study may assist clinical settings in diagnosing sarcopenia through blood analysis. This is not only effective, but also economical in comparison to physical performance tests. Further researches should aim towards establishing analytical validation of the proposed biomarker as well as qualification of the biomarker.

Several limitations in the present study should be noted. First, the study is a cross-sectional study. Thus, a causal relationship between PPIX and sarcopenia cannot be established. Long term observations are required to validate the relationship. Second, information such as participants’ medical histories and education levels were based on self-reported response to questionnaires. The effects of recall bias and other unknown errors may cause distortion in results. Third, the design of the current study may have been subjected to selection bias. This may lead to inaccurate representation of the relationship. Lastly, participants’ cognitive status [50], drug consumption [51], and nutritional status [52] were crucial factors of sarcopenia that were not taken into account. Thus, the effects of confusion bias could not be overlooked.

## Conclusion

In conclusion, our findings suggested strong correlations between PPIX and sarcopenia among both sexes. We also discovered significant associations under the state of low serum iron concentrations in non-anemic participants. Although further studies are required to validate the underlying mechanisms, the concentration of PPIX may be a valuable indicator for assessing the risk of sarcopenia in clinical settings.

## Supplementary Information

**Additional file 1 Table S1**. Characteristics of study participants. **Table S2.** Association between quartiles of protoporphyrin IX and components of sarcopenia. **Table S3.** Association between components of sarcopenia and protoporphyrin IX

## Data Availability

The data sets generated and/or analyzed during the current study are available from the NHANES repository, https://www.cdc.gov/nchs/nhanes/index.htm.

## Abbreviations

ANOVA: analysis of variance
ASM: appendicular skeletal muscle mass
AUROC: area under receiver operating characteristic
BMI: body mass index
CHD: Coronary heart disease
CHF: Congestive heart failure
CT: Computed tomography
CI: Confidence interval
DXA: Dual energy X-ray absorptiometry
EWGSOP: European Working Group on Sarcopenia in Older People
HO-1: Heme oxygenase-1
IRB: Institutional Review Board
IQR: Interquartile range
MEC: Mobile Examination Center
MRI: magnetic resonance imaging
NCHS: National Center for Health Statistics
NHANES: National Health and Nutrition Examination Survey
OD: Odds ratio
PPIX: Protoporphyrin IX
Q: Quartile
RBC: Red blood cells
ROC: Receiver operating characteristic
SD: Standard deviation
SMI: Skeletal muscle index

## References

[1] Cruz-Jentoft AJ, Sayer AA (2019). Sarcopenia. Lancet.

[2] Tsekoura M, Kastrinis A, Katsoulaki M, Billis E, Gliatis J (2017). Sarcopenia and its impact on quality of life. Adv Exp Med Biol.

[3] Walston JD (2012). Sarcopenia in older adults. Curr Opin Rheumatol.

[4] Xue QL, Walston JD, Fried LP, Beamer BA (2011). Prediction of risk of falling, physical disability, and frailty by rate of decline in grip strength: the women's health and aging study. Arch Intern Med.

[5] Moorthi RN, Avin KG (2017). Clinical relevance of sarcopenia in chronic kidney disease. Curr Opin Nephrol Hypertens.

[6] Cruz-Jentoft AJ, Bahat G, Bauer J, Boirie Y, Bruyere O, Cederholm T (2019). Sarcopenia: revised European consensus on definition and diagnosis. Age Ageing.

[7] McKee A, Morley JE, Matsumoto AM, Vinik A (2017). Sarcopenia: An Endocrine Disorder?. Endocr Pract.

[8] Kwon YN, Yoon SS (2017). Sarcopenia: neurological point of view. J Bone Metab.

[9] Beaudart C, Sanchez-Rodriguez D, Locquet M, Reginster JY, Lengele L, Bruyere O. Malnutrition as a Strong Predictor of the Onset of Sarcopenia. Nutrients. 2019;11(12):2883. 10.3390/nu11122883. 10.3390/nu11122883 PMC6950107 31783482

[10] Ali S, Garcia JM (2014). Sarcopenia, cachexia and aging: diagnosis, mechanisms and therapeutic options - a mini-review. Gerontology..

[11] Wall BT, Dirks ML, van Loon LJ (2013). Skeletal muscle atrophy during short-term disuse: implications for age-related sarcopenia. Ageing Res Rev.

[12] Calvani R, Joseph AM, Adhihetty PJ, Miccheli A, Bossola M, Leeuwenburgh C, et al. Mitochondrial pathways in sarcopenia of aging and disuse muscle atrophy. Biol Chem. 2013;394(3):393–414. 10.1515/hsz-2012-0247. 10.1515/hsz-2012-0247 PMC3976204 23154422

[13] Leduc-Gaudet JP, Picard M, St-Jean Pelletier F, Sgarioto N, Auger MJ, Vallee J (2015). Mitochondrial morphology is altered in atrophied skeletal muscle of aged mice. Oncotarget..

[14] Marzetti E, Calvani R, Cesari M, Buford TW, Lorenzi M, Behnke BJ, et al. Mitochondrial dysfunction and sarcopenia of aging: from signaling pathways to clinical trials. Int J Biochem Cell Biol. 2013;45(10):2288–301. 10.1016/j.biocel.2013.06.024. 10.1016/j.biocel.2013.06.024 PMC3759621 23845738

[15] Aiken J, Bua E, Cao Z, Lopez M, Wanagat J, McKenzie D (2002). Mitochondrial DNA deletion mutations and sarcopenia. Ann N Y Acad Sci.

[16] Bani Hassan E, Vogrin S, Hernandez Vina I, Boersma D, Suriyaarachchi P, Duque G (2020). Hemoglobin levels are low in Sarcopenic and Osteosarcopenic older persons. Calcif Tissue Int.

[17] Cesari M, Penninx BW, Lauretani F, Russo CR, Carter C, Bandinelli S (2004). Hemoglobin levels and skeletal muscle: results from the InCHIANTI study. J Gerontol A Biol Sci Med Sci.

[18] Hirani V, Naganathan V, Blyth F, Le Couteur DG, Seibel MJ, Waite LM (2016). Low hemoglobin concentrations are associated with sarcopenia, physical performance, and disability in older Australian men in cross-sectional and longitudinal analysis: the Concord health and ageing in men project. J Gerontol A Biol Sci Med Sci.

[19] Sachar M, Anderson KE, Ma X (2016). Protoporphyrin IX: the good, the bad, and the ugly. J Pharmacol Exp Ther.

[20] Lin YH, Chang HM, Chang FP, Shen CR, Liu CL, Mao WY, et al. Protoporphyrin IX accumulation disrupts mitochondrial dynamics and function in ABCG2-deficient hepatocytes. FEBS Lett. 2013;587(19):3202–9. 10.1016/j.febslet.2013.08.011. 10.1016/j.febslet.2013.08.011 23954234

[21] Jangid AP, John PJ, Yadav D, Mishra S, Sharma P (2012). Impact of chronic lead exposure on selected biological markers. Indian J Clin Biochem.

[22] National Center for Health Statistics (NCHS). National Health and Nutrition Survey Data. (1999–2000). Available from: https://wwwn.cdc.gov/nchs/nhanes/search/datapage.aspx?Component=Laboratory&CycleBeginYear=1999. [accessed March 2020].

[23] National Center for Health Statistics (NCHS). National Health and Nutrition Survey Data. (2001–2002). Available from: https://wwwn.cdc.gov/nchs/nhanes/search/datapage.aspx?Component=Laboratory&CycleBeginYear=2001. [accessed March 2020].

[24] Chaparro CM, Suchdev PS (2019). Anemia epidemiology, pathophysiology, and etiology in low- and middle-income countries. Ann N Y Acad Sci.

[25] National Center for Health Statistics (NCHS). National Health and Nutrition Examination Survey Questionnaire (or Examination Protocol, or Laboratory Protocol). (1999–2000). Available from: https://wwwn.cdc.gov/nchs/nhanes/continuousnhanes/manuals.aspx?BeginYear=1999. [accessed March 2020].

[26] National Center for Health Statistics (NCHS). National Health and Nutrition Examination Survey Questionnaire (or Examination Protocol, or Laboratory Protocol). (2001–2002). Available from: https://wwwn.cdc.gov/nchs/nhanes/continuousnhanes/manuals.aspx?BeginYear=2001. [accessed March 2020].

[27] Abdalla PP, Dos Santos CA, Dos Santos AP, Venturini ACR, Alves TC, Mota J (2020). Cut-off points of knee extension strength allometrically adjusted to identify sarcopenia risk in older adults: a cross-sectional study. Arch Gerontol Geriatr.

[28] Gould H, Brennan SL, Kotowicz MA, Nicholson GC, Pasco JA (2014). Total and appendicular lean mass reference ranges for Australian men and women: the Geelong osteoporosis study. Calcif Tissue Int.

[29] Fung FY, Koh YLE, Malhotra R, Ostbye T, Lee PY, Shariff Ghazali S, et al. Prevalence of and factors associated with sarcopenia among multi-ethnic ambulatory older Asians with type 2 diabetes mellitus in a primary care setting. BMC Geriatr. 2019;19(1):122. 10.1186/s12877-019-1137-8. 10.1186/s12877-019-1137-8 PMC6489356 31035928

[30] Du Y, Wang X, Xie H, Zheng S, Wu X, Zhu X (2019). Sex differences in the prevalence and adverse outcomes of sarcopenia and sarcopenic obesity in community dwelling elderly in East China using the AWGS criteria. BMC Endocr Disord.

[31] Nipp RD, Fuchs G, El-Jawahri A, Mario J, Troschel FM, Greer JA (2018). Sarcopenia is associated with quality of life and depression in patients with advanced cancer. Oncologist.

[32] Steffl M, Bohannon RW, Petr M, Kohlikova E, Holmerova I (2015). Relation between cigarette smoking and sarcopenia: meta-analysis. Physiol Res.

[33] Studenski SA, Peters KW, Alley DE, Cawthon PM, McLean RR, Harris TB (2014). The FNIH sarcopenia project: rationale, study description, conference recommendations, and final estimates. J Gerontol A Biol Sci Med Sci.

[34] Yoshimura Y, Wakabayashi H, Nagano F, Bise T, Shimazu S, Shiraishi A (2020). Low hemoglobin levels are associated with sarcopenia, dysphagia, and adverse rehabilitation outcomes after stroke. J Stroke Cerebrovasc Dis.

[35] Dhillon RJ, Hasni S (2017). Pathogenesis and Management of Sarcopenia. Clin Geriatr Med.

[36] Kamel HK, Maas D, Duthie EH (2002). Role of hormones in the pathogenesis and management of sarcopenia. Drugs Aging.

[37] Sayer AA, Robinson SM, Patel HP, Shavlakadze T, Cooper C, Grounds MD (2013). New horizons in the pathogenesis, diagnosis and management of sarcopenia. Age Ageing.

[38] Gan Z, Fu T, Kelly DP, Vega RB (2018). Skeletal muscle mitochondrial remodeling in exercise and diseases. Cell Res.

[39] Jornayvaz FR, Shulman GI (2010). Regulation of mitochondrial biogenesis. Essays Biochem.

[40] Takanche JS, Kim JE, Han SH, Yi HK (2020). Effect of gomisin a on osteoblast differentiation in high glucose-mediated oxidative stress. Phytomedicine..

[41] Meyer JN, Hartman JH, Mello DF (2018). Mitochondrial toxicity. Toxicol Sci.

[42] MacGarvey NC, Suliman HB, Bartz RR, Fu P, Withers CM, Welty-Wolf KE (2012). Activation of mitochondrial biogenesis by heme oxygenase-1-mediated NF-E2-related factor-2 induction rescues mice from lethal Staphylococcus aureus sepsis. Am J Respir Crit Care Med.

[43] Mahrouf-Yorgov M, Augeul L, Da Silva CC, Jourdan M, Rigolet M, Manin S (2017). Mesenchymal stem cells sense mitochondria released from damaged cells as danger signals to activate their rescue properties. Cell Death Differ.

[44] Takanche JS, Lee YH, Kim JS, Kim JE, Han SH, Lee SW, et al. Anti-inflammatory and antioxidant properties of Schisandrin C promote mitochondrial biogenesis in human dental pulp cells. Int Endod J. 2018;51(4):438–47. 10.1111/iej.12861. 10.1111/iej.12861 28898431

[45] Chen KD, Huang KT, Lin CC, Weng WT, Hsu LW, Goto S, et al. MicroRNA-27b enhances the hepatic regenerative properties of adipose-derived mesenchymal stem cells. Mol Ther Nucleic Acids. 2016;5:e285. 10.1038/mtna.2015.55. 10.1038/mtna.2015.55 PMC4884788 26836372

[46] Chen KD, Hsu LW, Goto S, Huang KT, Nakano T, Weng WT, et al. Regulation of heme oxygenase 1 expression by miR-27b with stem cell therapy for liver regeneration in rats. Transplant Proc. 2014;46(4):1198–200. 10.1016/j.transproceed.2013.12.013. 10.1016/j.transproceed.2013.12.013 24815159

[47] Yu W, Zhang X, Wu H, Zhou Q, Wang Z, Liu R (2017). HO-1 Is Essential for Tetrahydroxystilbene Glucoside Mediated Mitochondrial Biogenesis and Anti-Inflammation Process in LPS-Treated RAW264.7 Macrophages. Oxid Med Cell Longev.

[48] Yu X, Han W, Wang C, Sui D, Bian J, Bo L (2018). Upregulation of Heme Oxygenase-1 by hemin alleviates sepsis-induced muscle wasting in mice. Oxidative Med Cell Longev.

[49] Khan A, Jamwal S, Bijjem KR, Prakash A, Kumar P (2015). Neuroprotective effect of hemeoxygenase-1/glycogen synthase kinase-3beta modulators in 3-nitropropionic acid-induced neurotoxicity in rats. Neuroscience.

[50] Peng TC, Chen WL, Wu LW, Chang YW, Kao TW (2020). Sarcopenia and cognitive impairment: a systematic review and meta-analysis. Clin Nutr.

[51] Campins L, Camps M, Riera A, Pleguezuelos E, Yebenes JC, Serra-Prat M (2017). Oral drugs related with muscle wasting and sarcopenia. A Review. Pharmacology.

[52] Bertschi D, Kiss CM, Beerli N, Kressig RW (2021). Sarcopenia in hospitalized geriatric patients: insights into prevalence and associated parameters using new EWGSOP2 guidelines. Eur J Clin Nutr.

