# Supplementary material for: Association between protoporphyrin IX and sarcopenia: a cross sectional study
Source: BMC Geriatr. 2021 Jun 26;21:384. doi: 10.1186/s12877-021-02331-6 (PMC8235857; doi:10.1186/s12877-021-02331-6)
Supplement: Supplementary file 1 — Additional file 1 Table S1. Characteristics of study participants. Table S2. Association between quartiles of protoporphyrin IX and components of sarcopenia. Table S3. Association between components of sarcopenia and protoporphyrin IX [file 12877_2021_2331_MOESM1_ESM.docx]

**Table S1. Characteristics of study participants**

| **Characteristics of study participants** | **Q1** | **Q2** | **Q3** | **Q4** | ***P* value** |
| --- | --- | --- | --- | --- | --- |
|  | **(n = 331)** | **(n = 284)** | **(n = 272)** | **(n = 285)** |  |
| **Continuous variables ^a^** | | | | | |
| **Age at screening** | 64.66 (9.81) | 64.05 (9.42) | 66.64 (9.96) | 66.99* (9.35) | <0.001 |
| **Body mass index**  **(kg/m^2^)** | 27.89 (4.70) | 28.03 (4.81) | 28.47 (5.17) | 28.79 (5.58) | 0.111 |
| **Protoporphyrin**  **(****μg/dL RBC)** | 35.45 (3.61) | 44.56* (2.28) | 53.83* (3.03) | 75.59* (18.84) | <0.001 |
| **Hemoglobin**  **(g/dL)** | 15.04 (1.23) | 14.72* (1.11) | 14.39* (1.16) | 13.95* (1.08) | <0.001 |
| **Comorbidity** | 0.55 (0.89) | 0.62 (0.93) | 0.71 (0.89) | 0.80* (1.01) | 0.006 |
| **Average peak force (Newtons)** | 309.81 (107.17) | 299.96 (107.48) | 274.32* (103.50) | 252.64* (89.75) | <0.001 |
| **Skeletal muscle index (kg/m^2^)** | 7.67 (1.30) | 7.53 (1.41) | 7.34* (1.38) | 7.09* (1.45) | <0.001 |
| **Gait speed (m/s)** | 1.02 (0.22) | 1.01 (0.23) | 0.97 (0.22) | 0.95* (0.22) | <0.001 |
| **Categorical variables ^b^ (%)** | | | | | |
| **Race/ethnicity** | 72 (21.8) | 61 (21.5) | 61 (22.4) | 74 (26.0) | 0.062 |
| **Gender** | 232 (70.1) | 165 (58.1) | 126 (46.3) | 103 (36.1) | <0.001 |
| **Smoking** | 218 (65.9) | 166 (58.5) | 148 (54.4) | 124 (43.5) | <0.001 |
| **Education level past high school** | 204 (61.6) | 157 (56.3) | 161 (59.4) | 153 (53.7) | 0.173 |
| **Arthritis** | 118 (35.6) | 98 (34.5) | 116 (42.6) | 119 (41.8) | 0.098 |
| **Congestive heart failure** | 7 (2.1) | 9 (3.2) | 13 (4.8) | 22 (7.7) | 0.005 |
| **Coronary heart disease** | 13 (3.9) | 21 (7.4) | 16 (5.9) | 26 (9.1) | 0.060 |
| **Angina** | 17 (5.1) | 24 (8.1) | 15 (5.5) | 20 (7.0) | 0.425 |
| **Heart attack** | 22 (6.6) | 15 (5.3) | 19 (7.0) | 25 (8.8) | 0.433 |
| **Stroke** | 1 (0.3) | 1 (0.4) | 3 (1.1) | 3 (1.1) | 0.485 |
| **Emphysema** | 4 (1.2) | 8 (2.8) | 11 (4.0) | 13 (4.6) | 0.075 |

BMI, body mass index; RBC, red blood cell; Q, quartile.

^a^ Values were expressed as mean (standard deviation)

^b^ Values in the categorical variables were expressed as number (%)

* Indicates protoporphyrin IX quartiles (Q2, Q3, Q4) were significantly different from Q1 (p < 0.05, ANOVA)

The percentages of race/ethnicity, gender, and smoking represent Mexican American, men, and smokers respectively.

|  | | **Components of sarcopenia** | | | | | | | | | |
| --- | --- | --- | --- | --- | --- | --- | --- | --- | --- | --- | --- |
|  | | **Low average peak force (Newtons)** | | **Low skeletal muscle index** | | | | **Low gait speed** | | | |
| **Models ^a^** | **Quartiles of protoporphyrin** | **OR**  **(95% CI)** | ***P***  **Value** | | **OR**  **(95% CI)** | | ***P***  **Value** | | **OR**  **(95% CI)** | | ***P***  **Value** |
| **Model ^a^ 1** | **Q2 v.s. Q1**  **Q3 v.s. Q1**  **Q4 v.s. Q1** | 0.896 (0.532, 1.510)  1.900 (1.198, 3.012)  2.874 (1.859, 4.443) | 0.681  0.006  <0.001 | 0.972 (0.605, 1.562)  1.194 (0.753, 1.894)  1.530 (0.988, 2.371) | | 0.907  0.452  0.057 | | 1.289 (0.844, 1.970)  1.564 (1.031, 2.373)  1.727 (1.150, 2.594) | | 0.240  0.035  0.008 | |
| **Model ^a^ 2** | **Q2 v.s. Q1**  **Q3 v.s. Q1**  **Q4 v.s. Q1** | 0.851 (0.488, 1.484)  1.396 (0.846, 2.305)  2.091 (1.300, 3.364) | 0.570  0.192  0.002 | 1.013 (0.624, 1.644)  1.088 (0.674, 1.756)  1.389 (0.877, 2.201) | | 0.960  0.731  0.161 | | 1.321 (0.839, 2.078)  1.180 (0.750, 1.856)  1.254 (0.805, 1.952) | | 0.229  0.474  0.316 | |
| **Model ^a^ 3** | **Q2 v.s. Q1**  **Q3 v.s. Q1**  **Q4 v.s. Q1** | 0.756 (0.428, 1.333)  1.386 (0.829, 2.316)  1.934 (1.174, 3.185) | 0.333  0.213  0.010 | 1.076 (0.582, 1.987)  1.504 (0.805, 2.811)  2.258 (1.172, 4.349) | | 0.816  0.201  0.015 | | 1.170 (0.726, 1.886)  1.077 (0.667, 1.740)  0.982 (0.608, 1.587) | | 0.519  0.762  0.942 | |

**Table S2. Association between quartiles of protoporphyrin IX and components of sarcopenia**

BMI, body mass index; CI, confidence interval; OR, odds ratio; Q, quartile.

^a^ Adjusted covariates:

Model 1 = Unadjusted

Model 2 = Model 1 + age, sex, race/ethnicity

Model 3 = Model 2 + BMI, comorbidities, smoking, education level, hemoglobin

**Table S3. Association between components of sarcopenia and protoporphyrin IX**

|  |  | **Components of sarcopenia** | | | | | |
| --- | --- | --- | --- | --- | --- | --- | --- |
|  |  | **Low average peak force (Newtons)** | | **Low skeletal muscle index** | | **Low gait speed** | |
|  | **Models ^a^** | **β^b^**  **(95% CI)** | ***P***  **Value** | **β^b^**  **(95% CI)** | ***P***  **Value** | **β^b^**  **(95% CI)** | ***P***  **Value** |
| **Low iron** | **Model ^a^ 1** | 0.232 (0.052, 0.412) | 0.012 | 0.197 (0.033, 0.361) | 0.019 | 0.141 (-0.023, 0.304) | 0.091 |
|  | **Model ^a^ 2** | 0.212 (0.025, 0.398) | 0.026 | 0.175 (0.008, 0.343) | 0.040 | 0.100 (-0.067, 0.267) | 0.236 |
|  | **Model ^a^ 3** | 0.171 (-0.04, 0.345) | 0.055 | 0.233 (0.061, 0.406) | 0.009 | 0.063 (-0.132, 0.258) | 0.525 |
| **Normal iron** | **Model ^a^ 1** | 0.120 (0.072, 0.167) | <0.001 | 0.028 (-0.022, 0.079) | 0.268 | 0.084 (0.039, 0.128) | 0.001 |
|  | **Model ^a^ 2** | 0.064 (0.015, 0.113) | 0.010 | 0.007 (-0.042, 0.056) | 0.785 | 0.036 (-0.009, 0.082) | 0.116 |
|  | **Model ^a^ 3** | 0.065 (0.017, 0.113) | 0.008 | 0.061 (0.007, 0.114) | 0.021 | 0.021 (-0.025, 0.067) | 0.377 |

BMI, body mass index; CI, confidence interval.

^a^ Adjusted covariates

Model 1 = Unadjusted

Model 2 = Model 1 + age, sex, race/ethnicity

Model 3 = Model 2 + BMI, comorbidity, smoking, education level, hemoglobin

^b^ β coefficients were interpreted as change of components of sarcopenia for each increase in protoporphyrin IX concentration
